# Immune, Oxidative, and Morphological Changes in the Livers of Tibetan Sheep after Feeding Resveratrol and β-Hydroxy-β-methyl Butyric Acid: A Transcriptome–Metabolome Integrative Analysis

**DOI:** 10.3390/ijms25189865

**Published:** 2024-09-12

**Authors:** Xuan Chen, Fengshuo Zhang, Sayed Haidar Abbas Raza, Zhenling Wu, Quyangangmao Su, Qiurong Ji, Tingli He, Kaina Zhu, Yu Zhang, Shengzhen Hou, Linsheng Gui

**Affiliations:** 1 College of Agriculture and Animal Husbandry, Qinghai University, Xining 810016, China; 17318583359@163.com (X.C.); zhangfengshuo1997@163.com (F.Z.); w15165514762@163.com (Z.W.); qyam2737@163.com (Q.S.); jqr1960742393@163.com (Q.J.); 15297129283@163.com (T.H.); 15291938937@163.com (K.Z.); iszhangyu06@163.com (Y.Z.); qhdxhsz@163.com (S.H.); 2 Guangdong Provincial Key Laboratory of Food Quality and Safety, South China Agricultural University, Guangzhou 510642, China; haiderraza110@scau.edu.cn

**Keywords:** Tibetan sheep, transcriptomics, metabolomics, resveratrol, β-Hydroxy-β-methyl butyric acid

## Abstract

This study investigated the effects of dietary resveratrol (RES) and β-Hydroxy-β-methyl butyric acid (HMB) on immune, oxidative, and morphological changes in the livers of Tibetan sheep using transcriptomics and metabolomics. One hundred and twenty male Tibetan lambs of a similar initial weight (15.5 ± 0.14 kg) were randomly divided into four groups with thirty lambs per treatment: (1) H group (basal diet without RES or HMB); (2) H-RES group (1.5 g/day of RES); (3) H-HMB group (1250 mg/day of HMB); (4) H-RES-HMB group (1.5 g/day of RES and 1250 mg/day of HMB). The experiment was conducted for 100 days, including a pre-test period of 10 days and a formal period of 90 days. The results showed significantly increased concentrations of glutathione peroxidase, superoxide dismutase, and IgM in the H-RES-HMB group (*p* < 0.05), while the malondialdehyde levels were significantly decreased (*p* < 0.05). The glycolytic indices including creatinine kinase (CK), malate dehydrogenase (MDH), and succinate dehydrogenase (SDH) were significantly increased in the H-RES-HMB group compared with the others (*p* < 0.05). A histological analysis showed that the hepatic plate tissue in the H-RES-HMB group appeared normal with multiple cells. The transcriptomic analysis showed that the expression of genes associated with the calcium signaling pathway (*MYLK2*, *CYSLTR2*, *ADCY1*, *HRH1*, *ATP2B2*, *NOS2*, *HRC*, *ITPR1*, and *CAMK2B*) and the NF-κB signaling pathway (*BCL2* and *CARD14*) in the H-RES-HMB group were upregulated. The key differential metabolites (d-pyroglutamic acid, DL-serine, DL-threonine, fumarate, and glyceric acid) were enriched in the pathways associated with D-amino acid metabolism, the citrate cycle (TCA cycle), and carbon metabolism. The combined transcriptomic and non-targeted metabolomic analyses showed the co-enrichment of differential genes (*NOS2* and *GLUD1*) and metabolites (fumarate) in arginine biosynthesis-regulated glycolytic activity, whereas the differential genes (*ME1*, *SCD5*, *FABP2*, *RXRG*, and *CPT1B*) and metabolites (Leukotriene b4) co-enriched in the PPAR signaling pathway affected the immune response by regulating the PI3K/AKT and cGMP/PKG signaling. In conclusion, the dietary RES and HMB affected the hepatic antioxidant capacity, immune response, and glycolytic activity through modulating the transcriptome (*BCL2*, *CAMK2B*, *ITPR1*, and *IL1R1*) and metabolome (DL-serine, DL-threonine, fumaric acid, and glycolic acid).

## 1. Introduction

Tibetan sheep represent the most abundant livestock on the Qinghai–Tibet Plateau, mainly distributed at altitudes of over 3000 m [1]. However, Tibetan sheep face numerous challenges in this environment, including prolonged cold, low oxygen levels, and intense ultraviolet radiation, resulting in slow growth and poor reproductive performance [2]. Therefore, it is necessary to explore a feeding strategy, which can improve the feeding efficiency and feed-to-gain ratio and enhance the overall fattening of the sheep, yielding significant economic benefits [3].

Resveratrol (RES) is a polyphenolic phytotoxin extracted from He Shou Wu, grapes, peanuts, and other plants [4] that has anti-inflammatory, antioxidant, and anticancer properties [5]. In addition, RES has a wide range of biological and pharmacological activities [6]. It not only regulates antioxidant-related genes and counteracts oxidative stress [7] but also modulates various cellular processes in response to exogenous stimuli. β-Hydroxy-β-methyl butyric acid (HMB) is an intermediate product of leucine metabolism [8] that has biological effects such as the promotion of muscle protein synthesis and the inhibition of protein degradation; only about 5% of dietary leucine is oxidized to HMB [9]. Previous experimental studies have demonstrated that RES significantly reduced the expression levels of the genes related to the inflammatory response (TLR4, NF-κB, and c-jun) and attenuated the systemic, as well as hepatic inflammatory injuries caused by LPS in goats [10]. In addition, RES was found to significantly increase the body weights of calves, while reducing free fatty acid (FFA) and insulin (INS) levels in the serum and increasing the levels of serum growth hormone (GH), epidermal growth factor (EGF)/insulin-like growth factor-1 (IGF-I), and immunoglobulin A (IgA) [11]. It has also been found that the addition of the leucine metabolite HMB to sheep lymphocytes in an in vitro culture enhanced their immune function [12].

The liver is the largest parenchymal organ and digestive gland in mammals, which participate in many physiological processes, such as metabolism, detoxification, excretion, coagulation, and immunity [13]. Through promoting the lipid metabolism, the liver regulated the gluconeogenesis [14]. Hepatic sinusoidal cells are also responsible for the removal of harmful metabolites and foreign microorganisms, thereby maintaining the immune homeostasis [15]. Hepatic injure led to metabolic disorders, thus endangering organismal health [16]. The morphology of the liver tissue is considered a suitable indicator of nutritional status and metabolic function in animals. The higher the antioxidant capacity of the liver, the stronger the intracellular oxidative defense system [17]. The immune system represents the “army” that protects the body, with the functions of defense, surveillance, and maintaining stability, and specific indicators can be used to reflect the strength of the immune function and its ability to maintain the overall health of the animal body [18]. Therefore, a better understanding of the mechanism of RES and HMB supplementation in ruminant on the liver could contribute to the development of economically beneficial feeding strategies.

Multiomics association analysis is increasingly used for the analysis of complex traits in the exploration of the underlying molecular mechanisms in Tibetan sheep livers. Transcriptomics allows the identification of differentially expressed genes, while metabolomics identifies the changes in metabolites; an analysis of the association between the two datasets results in the determination of key gene targets, metabolites, and metabolic pathways. Therefore, this study employed an integrative approach combining transcriptomics and metabolomics to explore the impact of dietary RES and HMB supplementation on hepatic morphology, oxidative stress, and immune responses in Tibetan sheep.

## 2. Results

### 2.1. Determination of Antioxidant and Immune Indicators

Among the antioxidant indices, the levels of both GSH-PX and SOD were significantly higher in the H-RES-HMB group compared with the H, H-RES, and H-HMB groups (*p* < 0.05), while the MDA contents were significantly higher in the H, H-RES, and H-HMB groups relative to the H-RES-HMB group (*p* < 0.05). In terms of the immune indices, the IgM concentrations in the H-RES-HMB group were markedly raised compared with those in the H and H-RES groups (*p* < 0.05), while the glycolytic indices CK, MDH, and SDH in the H-RES-HMB group were significantly higher than those in the H, H-RES, and H-HMB groups (*p* < 0.05). The LDH levels were markedly raised in the H and H-HMB groups relative to the H-RES-HMB group (*p* < 0.05), while the LA contents were significantly higher in the H, H-RES, and H-HMB groups relative to those in the H-RES-HMB group (*p* < 0.05) (Table 1).

### 2.2. Histological Analysis of the Liver

**Figure 1:**
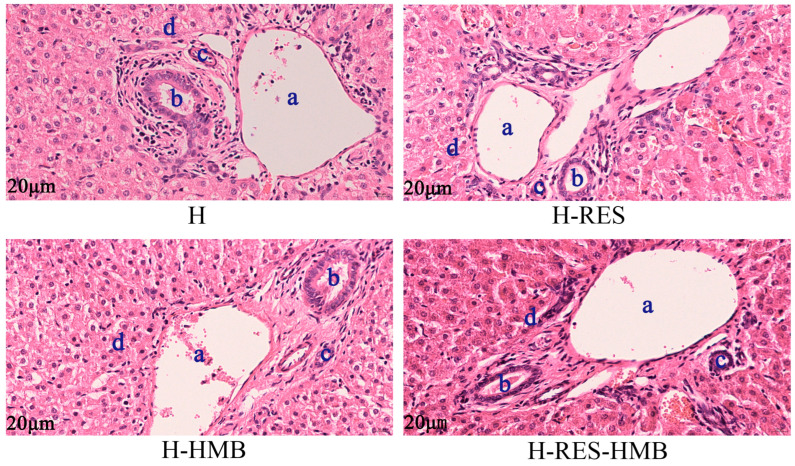
Frozen sections of liver tissue. Group H was the basal diet, the H-RES group was the basal diet + resveratrol (1.5 g/day), H-HMB was the basal diet + β-Hydroxy-β-methyl butyric acid (1250 mg/day), and H-RES-HMB was the basal diet + resveratrol (1.5 g/day) + β-Hydroxy-β-methyl butyric acid (1250 mg/day), with HE staining at 400×. a: interlobular vein; b: interlobular bile duct; c: interlobular artery; and d: hepatic sinusoids.

As shown in Figure 1, the histological structures of the liver tissue in the H group, the H-RES group, the H-HMB group, and the H-RES-HMB group were arranged radially with the central vein as the center to form a liver plate, with the anastomosis of the liver plates into a labyrinth-like structure. In contrast, the liver plates of the H and H-RES groups were separated from each other, with a loose distribution of the hepatocytes, while plasma was seen in the central veins of the H-HMB group, with extrahepatic sinusoidal dilation and congestion and a small amount of hepatocyte ballooning. In addition, the overall structure of the liver tissue in the H, H-RES, and H-HMB groups was mildly abnormal, with only the H-RES-HMB group showing a normal liver plate, which was not only matching but also had very full cells.

### 2.3. Identification of Differentially Expressed Genes and Functional Enrichment Analysis

**Figure 2:**
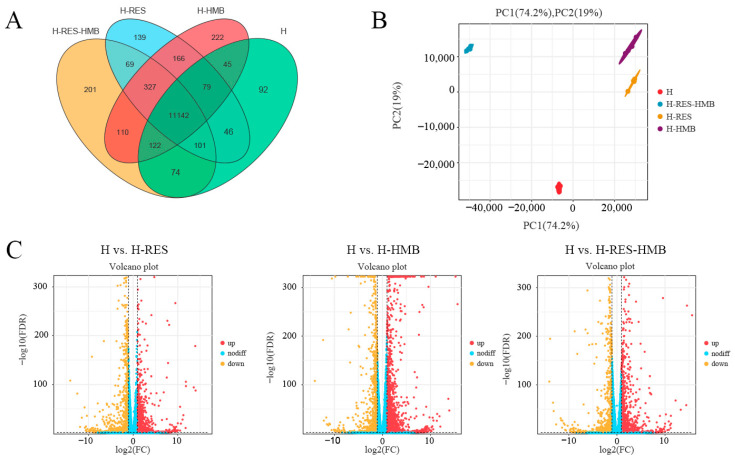
(**A**) Venn diagram, with the overlaps representing the number of genes shared between groups and the non-overlaps representing the genes unique to each group. (**B**) PCA plots for the comparison of samples within and between groups, analyzing the goodness of repeatability of the samples within a group versus the degree of variability of the samples between groups. (**C**) H vs. H-RES, H vs. H-HMB, H vs. H-RES-HMB: three groups of volcano plots of the DEGs; non-diff: non-differentially expressed genes.

There were 92 genes specific to the H group, with 139 in the H-RES group, 222 in the H-HMB group, and 201 in the H-RES-HMB group; and all four groups expressed 11,142 specific genes (Figure 2A). The PCA showed that 74.2% of the variance was explained by PC1 and 19% by PC2, with the two principal components covering 93.2% of the variance, and with a clear separation of the H-HMB and H-RES groups in comparison with the other two groups (Figure 2B). It can be seen that the confidence intervals for the H-RES-HMB group are larger and the kernel density is more concentrated, indicating the reliability and reproducibility of the data. To provide a better understanding of the effects of the addition of H-RES and H-HMB on the Tibetan sheep livers, the liver transcriptome was analyzed using RNA-seq. After high-throughput sequencing and filtration of the raw reads for quality control, 15.73, 14.93, 15.83, and 15.73 million high-quality clean reads were obtained for the H, H-RES, H-HMB, and H-RES-HMB sheep, respectively (Supplementary Table S1). The percentage of clean reads mapped to the sheep reference genome was between 98.50 and 99.50%. For each sample, >91% of the reads were mapped to the reference genome (Oar. v4.03). The default multiplicity of the difference adjustment range was greater than 2, and the significance threshold adjustment range was less than 0.05. Overall, 1808 DEGs were identified in the H vs. H-RES, H vs. H-HMB, and H vs. H-RES-HMB comparisons (Supplementary Table S2), with the results presented in a volcano plot (Figure 2C). The H vs. H-RES comparison yielded 2773 DEGs, including 1683 upregulated and 1090 downregulated genes; the H vs. H-HMB comparison yielded 3532 DEGs, including 2360 upregulated and 1172 downregulated genes; and the H vs. H-RES-HMB comparison identified 2297 DEGs, of which 1454 were upregulated and 843 were downregulated.

**Figure 3:**
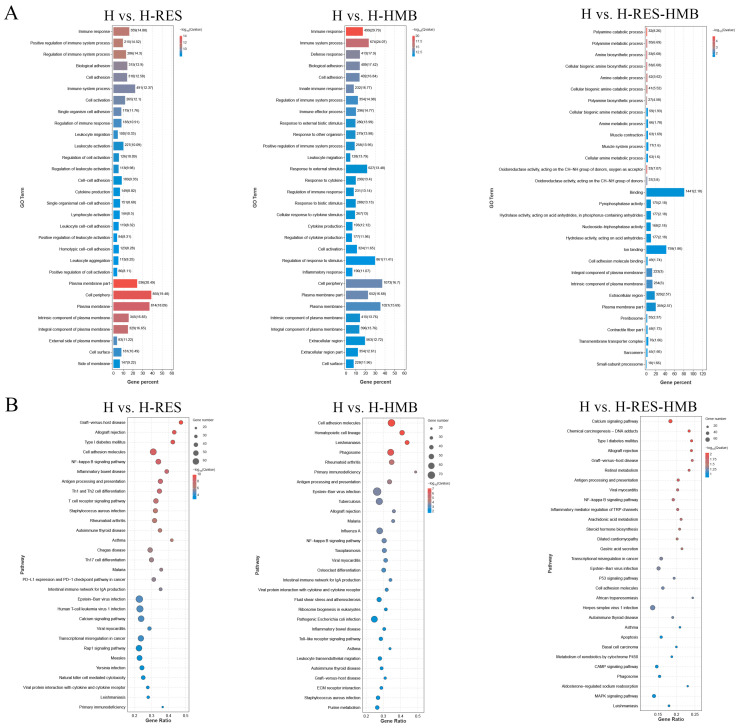
Functional enrichment analysis of the livers of H, H-RES, H-HMB, and H-RES-HMB Tibetan sheep. (**A**) GO enrichment bar chart to enrich and classify differential genes; the ordinate indicates the GO terms. (**B**) Bubble diagram of the top 30 KEGG pathway enrichments; the ordinate indicates the pathways.

The functions of the DEGs were explored using a functional enrichment analysis (Figure 3A). The GO analysis identified significant DEG enrichment in 67 terms, including 27 in the biological process (BP) category, 17 in the molecular function (MF) category, and 23 in the cellular component (CC) category. The enrichment was especially seen in terms associated with growth and development, including multicellular organismal process, metabolic processes, developmental processes, and cell proliferation and growth (the BP category); antioxidant activity, molecule function regulator, and catalytic activity (the MF category); and cell parts and organelles (the CC category). The KEGG enrichment results showed that the DEGs from the three groups were mainly enriched in the pathways related to human diseases, metabolism, organismal systems, and environmental information processing. Two pathways in particular showed the enrichment of multiple DEGs. In the NF-κB signaling pathway, the enriched DEGs were *GADD45B*, *BCL2*, *IL1R1*, and *CARD14*, while, in the calcium signaling pathway, the enriched genes were *MYLK2*, *CYSLTR2*, *ADCY1*, *HRH1*, *ATP2B2*, *NOS2*, *HRC*, *ITPR1*, and CAMK2B (Figure 3B and Supplementary Table S3).

**Figure 4:**
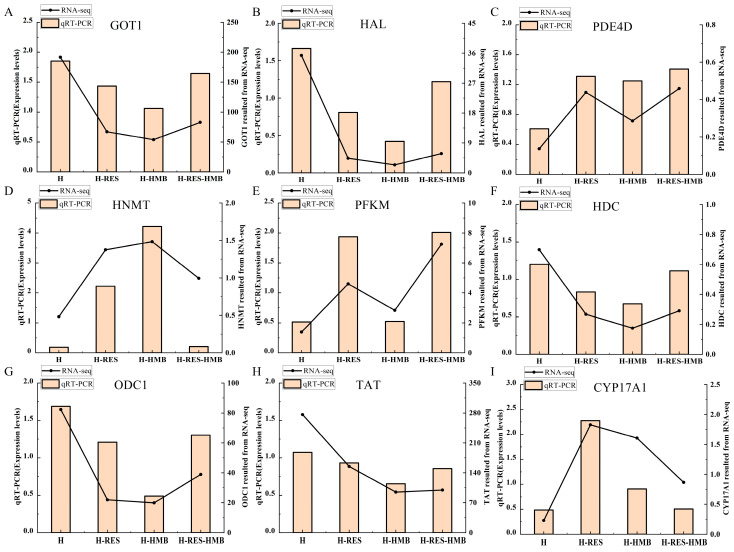
Confirmation of the expression patterns of the nine selected genes via the qRT-PCR. The qRT-PCR results were consistent with the RNA-seq data. (**A**) *GOT1*; (**B**) *HAL*; (**C**) *PDE4D*; (**D**) *HNMT*; (**E**) *PFKM*; (**F**) *HDC*; (**G**) *ODC1*; (**H**) *TAT*; and (**I**) *CYP17A1*.

To evaluate the reliability of the RNA-seq results, nine genes were selected and their expression levels were verified using a qRT-PCR. All the mRNA levels were consistent with the RNA-seq results, indicating that the RNA-seq data were reliable (Figure 4).

### 2.4. Metabolomic Profiling Based on LC-MS/MS

**Figure 5:**
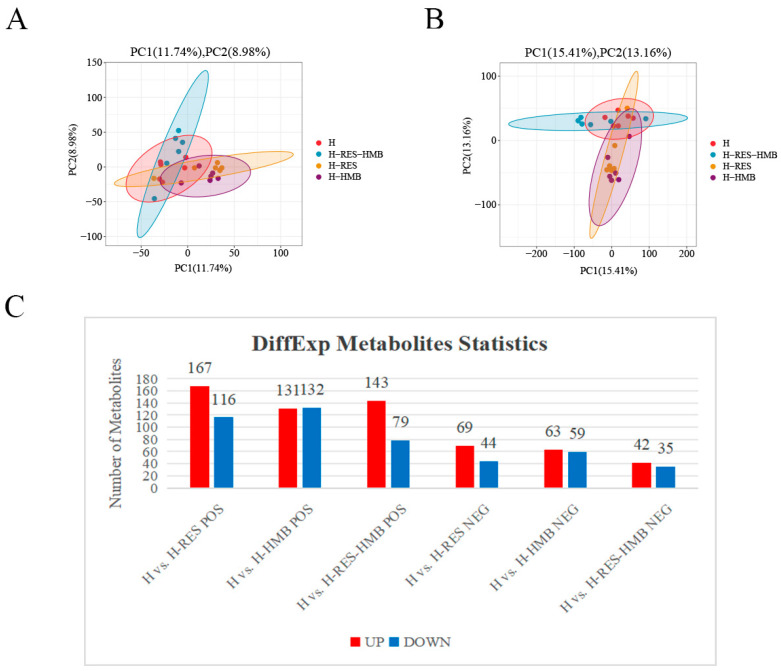
LC-MS/MS analysis of livers for the H vs. H-RES, H vs. H-HMB, and H vs. H-RES-HMB sheep. (**A**) Positive on the PCA score plot for the three groups. (**B**) Negative on the PCA score plot for the three groups. (**C**) Number of up-/downregulated metabolites of different compared groups in POS and NEG modes.

**Figure 6:**
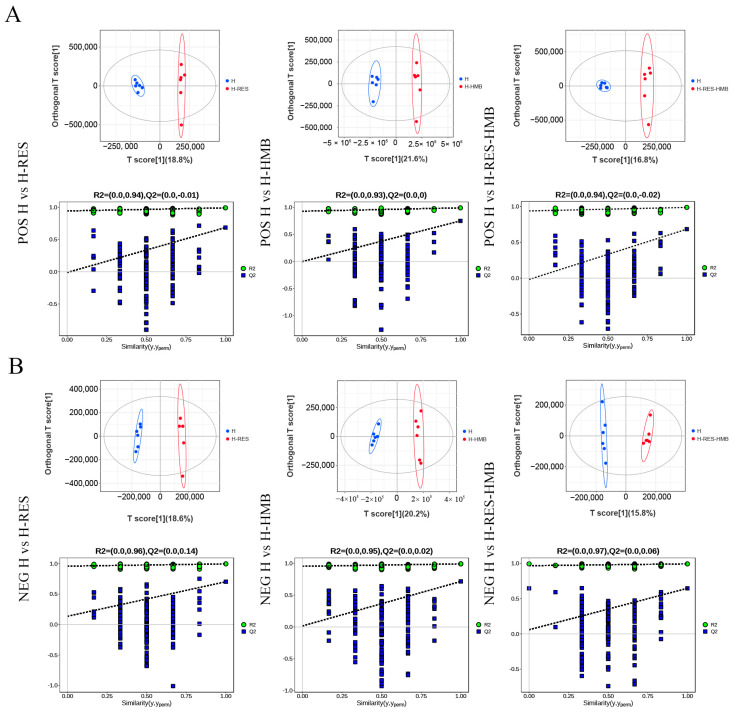
(**A**) Positive on the OPLS-DA model score plots for the three groups of samples. (**B**) Negative on the OPLS-DA model score plots for the three groups of samples. Red and blue represent different groups. The two dotted lines represent the regression lines of R2Y and Q2, respectively.

An LC-MS/MS analysis was used to investigate the differences in the metabolite compositions in the Tibetan sheep livers following different treatments. The PCA results showed that the three groups, including the quality control samples, were clearly separated in the principal component (PC)1 × PC2 score plot (Figure 5A,B). Furthermore, the OPLS-DA showed significant differences in the metabolites in the livers of the Tibetan sheep from the three groups (Figure 6A,B), thus demonstrating the accuracy and reproducibility of the assay using the LC-MS/MS method. A total of 1080 differential metabolites were identified (615 in the positive mode and 465 in the negative mode, Figure 5C and Supplementary Table S4), which were used for the multivariate analyses of the liver metabolites. This showed that 396 differential metabolites were significantly altered in the H-RES group, of which 236 were upregulated and 160 were downregulated. In the H-HMB group, 385 differential metabolites showed a significant change, of which 194 were upregulated and 191 were downregulated, while 299 differential metabolites were significantly altered in the H-RES-HMB group, with 185 being upregulated and 114 downregulated.

**Figure 7:**
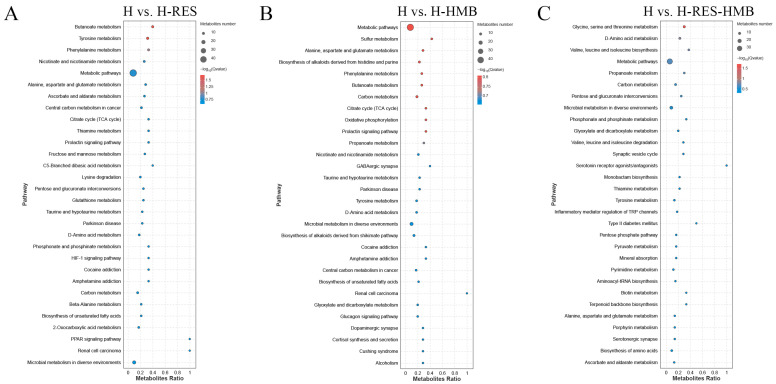
Bubble diagram of top 20 KEGG pathway enrichment in (**A**) H vs. H-RES, (**B**) H vs. H-HMB, and (**C**) H vs. H-RES-HMB comparisons. The ordinates indicate the pathways.

KEGG enrichment analysis was used to determine the biological mechanisms associated with the phenotypic changes. All the differential metabolites were co-enriched (*p* < 0.05) in 48 metabolic pathways. In the H vs. H-RES comparison, 110 differential metabolites were significantly annotated into 16 pathways, with most involved in butanoate metabolism; tyrosine metabolism; phenylalanine metabolism; nicotinate and nicotinamide metabolism; and alanine, aspartate, and glutamate metabolism. In the H vs. H-HMB comparison, 98 differential metabolites were significantly associated with 19 pathways (*p* < 0.05), most of which were involved in sulfur metabolism; alanine, aspartate, and glutamate metabolism; the biosynthesis of alkaloids derived from histidine and purine; phenylalanine metabolism; and butanoate metabolism, among others. In the H vs. H-RES-HMB comparison, 101 differential metabolites were significantly annotated in 13 pathways (*p* < 0.05), most of which were involved in glycine, serine, and threonine metabolism; D-amino acid metabolism; valine, leucine, and isoleucine biosynthesis; propanoate metabolism; and carbon metabolism (Figure 7 and Supplementary Table S5).

### 2.5. Integrative Analysis of the Transcriptome and Metabolome

**Figure 8:**
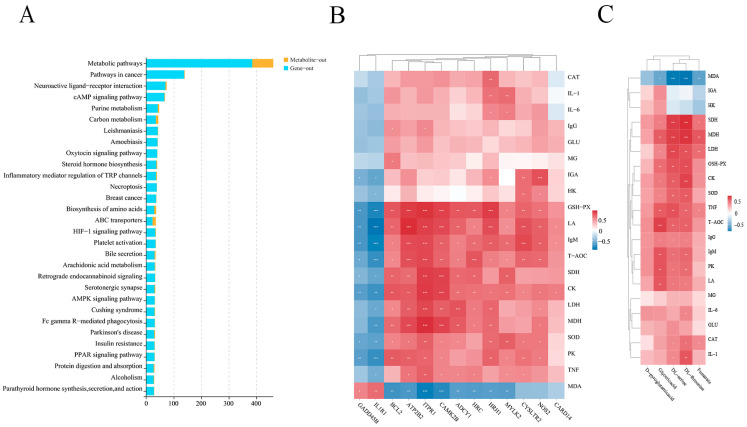
(**A**) Pathway correlation analysis histograms; (**B**) transcribed genes versus kit metrics heat map correlation analysis; and (**C**) metabolites versus kit metrics heat map correlation analysis. * *p* < 0.05, ** *p* < 0.01, *** *p* < 0.001.

To obtain more biological information from the transcriptomic and metabolomic analyses of the liver, the associations between the transcriptome and the metabolome were analyzed (Supplementary Table S6) (Figure 8A). This combined transcriptomic and non-targeted metabolomic analysis indicated the involvement of the pathways associated with the arginine biosynthesis and PPAR signaling pathway. Specifically, the DEGs *(NOS2* and *GLUD1*) and DAMs (fumarate) were co-enriched in the arginine biosynthesis, a pathway that can regulate the phenotypic features related to glycolytic activity through the TCA cycle, while the DEGs (*ME1*, *SCD5*, *FABP2*, *RXRG*, and *CPT1B*) and DAMs (leukotriene b4) were co-enriched in the PPAR signaling pathway, which affects the immune response through the regulation of the PI3K/AKT and cGMP/PKG signaling (Figure 8B). The relationships between the DEGs and the antioxidant, immune, and glycolytic indices showed that, in terms of the antioxidant indices, GSH-PX was positively correlated with the *HRH1*, *CAMK2B*, *ITPR1*, and *ATP2B2*, and MDA was negatively correlated with *CAMK2B* and *ITPR1*. Among the immune indices, IgM was positively associated with the DEGs (*CYSLTR2*, *ITPR1*, and *ATP2B2*). Figure 8C showed the relationships between the DAMs and the antioxidant, immune, and glycolytic indices, indicating that the antioxidant index MDA was negatively correlated with the DAMs DL-serine and DL-threonine, while, among the glycolytic indices, LDH was positively correlated with the DL-serine. SDH and MDH were positively correlated with the DL-serine and DL-threonine.

## 3. Discussion

The liver is covered with a plasma membrane and is rich in elastic fibers [19]. The mammalian liver lobules consist of various parts, including the central vein, hepatocytes, hepatic plates, sinusoids, perisinusoidal spaces, and null cells [20]. Previously, RES regulated the microbiota–gut–liver axis, in particular increasing the relative enrichment of *Bifidobacterium* in the intestinal tract, thereby alleviating the liver fibrosis induced by inorganic mercury [21]. Additionally, RES reduced α-amanitin-induced liver damage via modulating the *NFkB* and *LC3B* expression [22]. Here, the data in this present study were in accordance with the above results, in which dietary RES supplementation significantly contributed to the hepatic phenotype. It was noteworthy that the structure of the liver tissue in all groups exhibited universal disturbance. One possible explanation is that persistent exposure in a high altitude environment can especially cause hypobaric hypoxia, which results in damage to the cardiovascular, respiratory, musculoskeletal, reproductive, and metabolic organ systems [23].

Oxidative stress refers to a distortion in the redox balance of cells under a pathological condition, which results in damage to DNA, proteins, and lipids. Oxidative stress has been recognized as a key player in the process of tissue damage [24]. Currently, several natural extracts, including L-carnitine, coenzyme Q10, and allicin, have exhibited substantial protective effects against oxidative damage [25]. In this study, dietary RES and HMB supplementation alone or in combination significantly influenced the concentrations of GSH-PX and SOD. GSH-Px plays a crucial role in the conversion of lipid hydroperoxides to their corresponding alcohols, reducing free hydrogen peroxide to water, as well as reducing organic hydrogen peroxide [26]. SOD, an antioxidant enzyme located in the mitochondrial matrix, is responsible for scavenging locally produced free radicals, which are then converted into water [27]. One study found that the addition of RES significantly increased the activities of SOD and GSH-PX and decreased the levels of MDA [28]. Another study found that the addition of HMB increased the SOD activity and reduced the MDA concentrations in ruminants [29]. In this current study, the simultaneous administration of both RES and HMB resulted in significantly higher SOD and GSH-PX levels and a lower MDA concentration, whereas the single treatment with RES (1.5 g/day, 90 days) or HMB (1.25 g/day, 90 days) was inconsistent with the results of the previously mentioned studies, suggesting the synergistic effects of RES and HMB in maintaining a prooxidative–antioxidative balance in the livers of Tibetan sheep. As a natural phytoalexin, the RES was involved in the function of antioxidation and anti-inflammation. Through modulating the NF-κB signaling pathway, the RES diminished the immunity-related gene expression (IgM). The expressions of the antioxidant genes, including GSH-PX and SOD, were increased when supplemented with RES in the bovine embryos [30,31]. Similarly, HMB regulated the mTORC1 signaling pathway and NF-κB signaling pathway, which are closely associated with the immune function and oxidation state [32,33].

Glycolysis is the major glucose-consuming pathway for the production of energy and metabolites in most organisms [34]. The overexpression of LDH in lung cancer cells increased the inverse reaction of the TCA cycle, thereby promoting anaerobic glycolysis [35]. CK is mainly found in cells with rapid ATP regeneration, where it is involved in phosphocreatine shuttling, ATP regeneration, phagocytosis, glycolysis regulation, and neurotransmitter release [36]. MDH is a key enzyme in fat synthesis and is secreted by the liver; it is directly involved in the biosynthesis of long-chain fatty acids [37]. SDH plays a key role in aerobic respiration and is a major enzyme involved in the mitochondrial energy supply [38]. LA is mainly produced by the fermentation of glucose by lactic acid bacteria, and its content is usually positively associated with fermentation quality [39]. Previous research reported that RES converted phosphofructokinase (PFK) from an active tetrameric form to an inactive dimeric form, thus inhibiting PFK activity and reducing LA production [40]. The effects of HMB on glycolysis are not as well known, although one study found that the expression of Sox6 mRNA in the dorsal longissimus dorsi muscle of piglets was significantly increased in response to HMB and that it was specifically able to promote the conversion of enzymes [41]. In this study, the highest activities of CK, MDH, and LDH were observed in the RES-HBM group, suggesting that RES and HMB exhibited considerable protective effects on hepatic glycolysis.

In this study, the DEGs were enriched in the calcium signaling pathway and the NF-κB signaling pathway. Calcium (Ca^2+^) is an essential signaling molecule that participates in a wide range of biological functions, such as proliferation, differentiation, apoptosis, and the transcription of numerous genes [42]. NF-κB signaling plays a key role in liver ischemia-reperfusion injury (HIRI). Moreover, the NF-κB pathway also regulates the immune response via suppressing apoptosis [43]. In this current study, nine DEGs, including *MYLK2*, *CYSLTR2*, *ADCY1*, *HRH1*, *ATP2B2*, *NOS2*, *HRC*, *ITPR1*, and *CAMK2B*, were identified, which were enriched in the calcium signaling pathway. *CAMK2B* is involved in the pyruvate metabolic pathway. Pyruvate is not only an important metabolic substrate but also has antioxidant effects and, through the phosphorylation of *CAMK2B*, can enter the TCA cycle and participate in oxidative phosphorylation. In addition, CAMK2B plays a key role in cell adhesion and signaling and can repair neurons [44]. *ITPR1* is a glycoprotein that shows unique glycosylation and is associated with immune infiltration. It has been found that ITPR1 protects renal cancer cells against the action of natural killer cells by inducing autophagy [45]. Additionally, two DEGs, including *BCL2* and *CARD14*, were enriched in the NF-κB signaling pathway. The overexpression of BCL-2 reduces the production of oxygen free radicals and the subsequent formation of lipid peroxides. *BCL2* also increases the levels of intracellular antioxidants, such as glutathione (GSH), and elevates the NAD/NADH ratio, thus affecting the redox state of cells [46,47].

The liver is the central organ of metabolism and is involved in the metabolism of sugars, proteins, lipids, vitamins, hormones, and bilirubin; the secretion of bile; and the excretion of metabolites, drugs, and detoxification products [48]. Using untargeted metabolomics, three significant pathways were identified in the H vs. H-RES, H vs. H-HMB, and H vs. H-RES-HMB groups, namely, the TCA cycle, D-Amino acid metabolism, and carbon metabolism. In the TCA cycle pathway, the metabolite fumarate modulates tyrosine metabolism and arginine biosynthesis; the inhibition of tyrosine metabolism by the inhibitor dasatinib can influence the immune system, especially conventional T cells and natural killer cells [49]. Arginine biosynthesis can influence nutrient metabolism, stimulate insulin release, participate in non-specific immune and antioxidant responses, and improve disease resistance [50]. Interestingly, the metabolites fumarate and succinate have antagonistic effects in the TCA cycle, affecting oxidative phosphorylation, as well as ROS levels, leading to oxidative stress and a reduction in antioxidant capacity [51]. Moreover, fumarate has antioxidant, immunomodulatory, and neuroprotective properties and is useful in the treatment of other diseases [52]. The significant upregulation of DL-serine and DL-threonine metabolites was found in association with the D-Amino acid metabolic pathway. DL-serine is an α-amino acid with alanine substituted with a hydroxyl group at position 3 [53], while DL-Threonine is an essential amino acid that is particularly involved in protein synthesis and contributes to the construction of proteins, cell membranes, and neurotransmitter production [54]. Threonine has been found to immunomodulate gastrointestinal problems in sheep [55]. In this present study, it was found that the D-amino acid metabolic pathway was associated with the TCA cycle. DL-serine and DL-Threonine are racemic mixtures of isomers derived from the differentiation of their precursors, serine and threonine, and are produced by glycolysis, as well as through pyruvic acid metabolism into the TCA cycle to enhance glycolytic activity. In the carbon metabolism pathway, the metabolite glycerate is upregulated and enters the TCA cycle via phosphoenolpyruvate to regulate the glycolytic activity. Moreover, the carbon metabolic pathway provides the necessary carbon framework and energy for the synthesis of amino acids, proteins, and nucleic acids. It has been found that the metabolite glyceric acid, a tricarboxylic acid formed from the oxidation of glycerol, is an intermediate in the degradation of serine and phosphorylates to produce 3-phosphoglyceric acid, which can be further isomerized to sugars or further involved in glycolysis [56].

In this study, the metabolomic and transcriptomic profiles of liver tissues were comprehensively analyzed, showing that these genes and metabolites were enriched in the immune response, glycolysis, arginine biosynthesis, and PPAR signaling pathways. In the arginine biosynthesis pathway, the *NOS2* (DEG) and the fumarate (DAM) were significantly upregulated into the TCA cycle, which enhanced the glycolytic activity [57]. Moreover, this pathway promotes the upregulation of the DAM L-Glutamate through the *GLUD1* (DEG). Furthermore, l-Glutamate has been found to enhance antioxidant capacity [58]. The PPAR signaling pathway plays a crucial role in systemic cell metabolism, energy homeostasis, and the suppression of the immune response [59]. This pathway was found to be enriched with the significantly upregulated *SCD-1*, *ME1*, *CPT-1*, *RXRG*, and *FABP2*, as well as the Leukotriene b4. FABP2 is known to regulate 9-Cis-Retinoic acid. 9-Cis-Retinoic acid is a retinoic acid X receptor (RXR) high-affinity ligand that regulates allergic immune responses by decreasing IgE responses versus promoting IgA responses [60,61]. The metabolite 9-Cis-Retinoic acid, together with the differential metabolite Leukotriene b4, regulates the adipocytokine signaling pathway, and the resulting hormones and cytokines are able to regulate multiple downstream signaling pathways by binding to its receptor, thereby affecting energy metabolism and the immune response and other physiological processes [62]. Interestingly, the *RXRG* in the adipocytokine signaling pathway binds to specific DNA elements and promotes the upregulation of *SCD-1*, *ME1*, and *CPT1B*. Mechanistically, PPARγ can antagonize the activation of hepatic stellate cells during hypoxia and can be activated by regulating PI3K/AKT and cGMP/PKG signaling [63]. The exposure to a variety of microorganisms in the gastrointestinal tract promotes sustained B cell pool diversification and T cell-independent antibody production, including high levels of IgA [64]. This suggests that the pathway has a major impact on the immune response.

## 4. Materials and Methods

### 4.1. Ethics Statement

This experimental study was approved by the Institutional Animal Care and Use Committee of Qinghai University (license number: QUA-2020-0710; approval date: 10 June 2020).

### 4.2. Animal Feed and Sample Collection

One hundred and twenty healthy, two-month-old Tibetan lambs with a similar body weight (16.87 ± 0.31 kg) were randomly divided into 4 treatments, with 30 lambs in each treatment, 5 replicates in each group, and 6 lambs in each replicate. The 4 treatments were the basal diet (the H group), the basal diet + RES (1.5 g/day) (the H-RES group), the basal diet + HMB (1250 mg/day) (the H-HMB group), and the basal diet + RES (1.5 g/days) + HMB (1250 mg/day) (the H-RES-HMB group), respectively. The duration of the experiment was 100 days, including a pre-test period of 10 days and a formal period of 90 days. The lambs were kept in an enclosure that was sunny, sheltered, and dry, and equipped with a sports field for free movement. Before the start of the test, the enclosure was fully disinfected. Each group was fed twice a day (08:00 and 17:00) according to the experimental design. The animals had free access to water, with the drinking trough cleaned each day. Disinfection, immunization, and health care were carried out in accordance with the regulations of the sheep farm. At the end of the trial, the lambs were slaughtered on day 91 of the experimental period, and the liver tissue was collected. A portion of the tissue samples was snap-frozen in liquid nitrogen and preserved in a −80 °C freezer for RNA isolation, while the remaining tissue samples were fixed in 4% paraformaldehyde for sectioning (Table 2).

### 4.3. Determination of Antioxidant, Immune, and Glycolytic Indices in the Liver Tissues

After the selection of an appropriate amount of liver tissue, the tissue was washed in a pre-cooled phosphate-buffered saline (PBS, 0.02 mol/L) and weighed. The cleaned tissue was then homogenized in a tissue homogenizer (1 g of sample per 5 mL of PBS), followed by centrifugation (5000 rpm, 5 min), and the supernatant was retained. The antioxidant, immune, and glycolytic indices were then measured by an ELISA in the supernatants using the enzyme-linked immunosorbent assay kit (Enzyme Immunity Industry Co., Ltd., Nanjing, China). The antioxidant indices were glutathione peroxidase (GSH-PX), superoxide dismutase (SOD), total antioxidant capacity (T-AOC), catalase (CAT), and malondialdehyde (MDA), while the immune indices were IgA, IgG, IgM, TNF-α, IL-1β, and IL-6 and the glycolytic indices were lactate dehydrogenase (LDH), GLU, MG, hexokinase (HK), pyruvate kinase (PK), creatine kinase (CK), malate dehydrogenase (MDH), succinate dehydrogenase (SDH), and lactic acid (LA).

### 4.4. Liver Histological Analysis

The liver tissues were fixed in 4% paraformaldehyde and embedded in paraffin. The paraffin-embedded tissues were cut into 3 µm sections using a microtome, and the sections were stained with hematoxylin and eosin (H&E). The sections were evaluated and imaged under optical microscopy (Olympus corporation, Tokyo, Japan). The liver cross-sectional area was measured using Image-Pro Plus 6.0 software (Media Cybernetics, Bethesda, MD, USA).

### 4.5. RNA Sequencing (RNA-Seq) and Data Analysis

The total RNA was extracted from the liver tissue using TRIzol kits (Invitrogen, Waltham, MA, USA) following the manufacturer’s protocol. The RNA quality was assessed on an Agilent 2100 bioanalyzer (Agilent Technologies, Santa Clara, CA, USA) and checked using RNase-free agarose gel electrophoresis. After enrichment of the mRNA with polyA tails by magnetic beads with Oligo (dT), the mRNA was ultrasonicated. The first and second cDNA strands were synthesized using M-MuLV reverse transcriptase using fragmented mRNA as the template and random oligonucleotides as the primers. The purified double-stranded cDNA underwent end repair and poly(A) tail and sequencing adapter removal, and about 200 bp of cDNA was screened with AMPure XP beads and amplified by PCR. The PCR products were purified again with AMPure XP beads to obtain the library. The raw reads were filtered, and the clean reads were mapped to the reference sequence using HISAT 2.2.4 software [65]. The gene expression levels were calculated using the fragments per kilobase per million mapped reads (FPKM) method. Finally, the DESeq2 software was used to determine the differential RNA expression between the groups [66] using the criteria of *p* < 0.05|log-fold change| ≥ 2 to identify the differentially expressed genes (DEGs). The Gene Ontology (GO) and Kyoto Encyclopedia of Genes and Genomes (KEGG) enrichment analyses of the DEGs were performed using R based on the hypergeometric distribution. The GO terms and KEGG pathways with *p* < 0.05 were considered significantly enriched.

To ensure the quality of the sequencing, it was necessary to use strict criteria for assessing the quality of the library construction. The standards used were agarose gel electrophoresis for the evaluation of the RNAo integrity and the presence of DNA contamination, nanospectrophotometry for examining the RNA purity and concentration (OD260/280 and OD260/230 ratios), fluorometry (Qubit 2.0 fluorometer, Thermo Fisher, Waltham, MA, USA) for the quantification of the RNA concentration, and the 2100 bioanalyzer (Agilent, CA, USA) for the accurate determination of the RNA integrity.

### 4.6. Metabolite Extraction for LC-MS/MS Analysis

For the metabolite extraction, the freeze-dried samples were dissolved in methanol and concentrated by vacuum-drying after centrifugation. The samples were then dissolved in 80% 2-chlorobenzalanine and filtered (20 μL of each sample was used for quality control [QC]). The autosampler temperature was 8 °C. The analytes were eluted with a gradient of 0.1% formic acid in water (C) and 0.1% formic acid in acetonitrile (D) or 5 mM of ammonium formate in water (A) and acetonitrile (B) at a flow rate of 0.25 mL/min. After equilibration, 2 μL of the sample was injected, and the following increasing linear gradient of solvent B (*v*/*v*) was used: 0–1 min, 2% B/D; 1–9 min, 2–50% B/D; 9–12 min, 50–98% B/D; 12–13.5 min, 98% B/D; 13.5–14 min, 98-2% B/D; and 14–20 min, 2% D + the positive control. The ESI-MSn experiments were performed on a Thermo Q Exactive mass spectrometer (Thermo Fisher) with spray voltages of 3.8 and −2.5 kV in the positive and negative ion modes (14–17 min, 2% B-negative model), respectively. The sheath and auxiliary gases were set at 30 and 10 arbitrary units, respectively. The capillary temperature was 325 °C. The analyzer scanned over a mass range of 81–1000 *m*/*z* for a full scan at a mass resolution of 70,000. The data-dependent acquisition (DDA) MS/MS experiments were performed with an HCD scan. The normalized collision energy was 30 eV.

The metabolites were distinguished by a comparison of the *m*/*z* values of the precursor ions, retention times, and fragmentation patterns with the standards in a database compiled by Gene Denovo Biotechnology Co., Ltd., Guangzhou, China. The metabolic alterations among the groups were analyzed via principal component analysis (PCA) and (orthogonal) partial least-squares discriminant analysis (OPLS-DA) after the data preprocessing by mean centering (Ctr) and Pareto variance (Par) scaling, respectively. The differential metabolites were identified using a threshold of *p* < 0.05 in the *t*-tests, and the metabolites with a VIP ≥ 1 were considered differential between the groups. The metabolic pathways in which the identified differential metabolites were involved were analyzed using the KEGG database.

### 4.7. Integrative Analysis of the Metabolome and Transcriptome

The transcriptomic and metabolomic data were analyzed to determine the differences in gene expression and metabolite abundance. The associations between these differential genes and metabolites were assessed using a KEGG pathway analysis in which the differential genes were significantly enriched together with differentially abundant metabolites (DAMs). The associations between the pathways enriched in the genes and the metabolites were visualized using Cytoscape (V3.3.0) software. The relationships of the DEGs and DAMs to the phenotypic traits were analyzed using Pearson’s correlations.

### 4.8. Statistical Analysis

The experimental data were statistically analyzed using SPSS 26.0 software (IBM Corp., Armonk, NY, USA) using one-way ANOVA tests. The data are presented as the mean ± standard error of the mean, and *p*-values < 0.05 were considered statistically significant. The graphs were drawn with Origin 86 (2021).

### 4.9. Verification of RNA-Seq Results with Quantitative Reverse Transcription Polymerase Chain Reaction (qRT-PCR)

The mRNA expression of the DEGs was normalized against the levels of the housekeeping gene glyceraldehyde 3-phosphate dehydrogenase (GAPDH) in the respective samples. The primers used for this study were designed using an online primer design tool and are presented in Table 3. The relative expression levels of the genes were determined using the 2^−ΔΔCt^ method.

## 5. Conclusions

**Figure 9:**
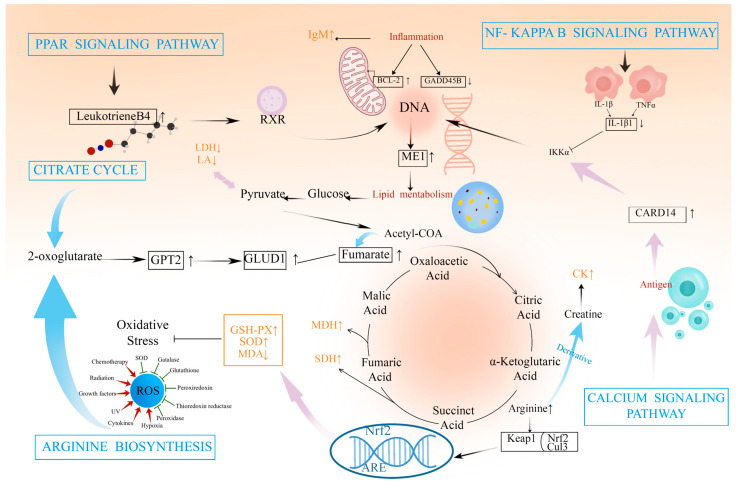
Map of the mechanisms by which the liver transcriptome is associated with the non-target metabolome.

In summary, the simultaneous addition of RES and HMB was found to affect the hepatic antioxidant capacity (GSH-PX and SOD), immune response (IgM), and glycolytic activity (LDH, CK, MDH, SDH, and LA) by regulating the gene expression (*BCL2*, *CAMK2B*, *ITPR1*, and *IL1R1*) and metabolism (DL-serine, DL-threonine, fumarate, and glyceric acid). Additionally, RES and HMB exhibited protective effects on the hepatic function via modulating the TCA pathway and carbon metabolism pathway, thereby contributing to the antioxidant capacity and immune response (Figure 9).

## Figures and Tables

**Table 1:** Determination of antioxidant indices, immunological indices, and glycolytic indices.

Items	Groups				*p*-Value
	H	H-RES	H-HMB	H-RES-HMB	
Antioxidant					
GSH-PX (Pmol/mL)	52.41 ± 0.99 ^b^	63.41 ± 1.49 ^b^	62.21 ± 2.31 ^b^	86.43 ± 6.20 ^a^	0.001
SOD (pg/mL)	8.40 ± 0.42 ^b^	9.32 ± 0.47 ^b^	10.29 ± 0.11 ^b^	13.40 ± 2.04 ^a^	0.048
T-AOC (u/mL)	10.16 ± 0.68	10.21 ± 0.63	11.76 ± 0.55	10.49 ± 1.06	0.406
CAT (ng/L)	147.19 ± 2.36	147.84 ± 4.84	148.17 ± 15.93	160.59 ± 3.00	0.651
MDA (Pg/mL)	10.34 ± 0.27 ^a^	8.68 ± 1.19 ^a^	9.87 ± 1.45 ^a^	4.35 ± 0.42 ^b^	0.009
Immunological					
IgA (μg/mL)	8.39 ± 0.88	9.18 ± 0.19	11.69 ± 1.50	10.40 ± 0.65	0.145
IgG (μg/mL)	886.11 ± 44.91	937.96 ± 108.60	962.04 ± 12.96	1071.29 ± 138.20	0.559
IgM (μg/mL)	13.30 ± 0.74 ^b^	15.14 ± 0.78 ^b^	15.99 ± 0.31 ^ab^	18.90 ± 1.46 ^a^	0.016
TNF-α (ng/L)	665.51 ± 10.01	689.87 ± 35.27	709.10 ± 102.59	523.21 ± 96.19	0.326
IL-1β (ng/L)	84.09 ± 3.81	85.86 ± 6.14	83.59 ± 0.91	74.49 ± 3.51	0.266
IL-6 (ng/L)	117.59 ± 12.05	133.25 ± 29.59	106.75 ± 35.87	87.87 ± 20.65	0.673
Glycolytic					
LDH (ng/L)	31.51 ± 1.42 ^a^	23.32 ± 4.71 ^ab^	27.14 ± 4.57 ^a^	13.50 ± 2.50 ^b^	0.038
GLU (μmol/L)	496.15 ± 40.53	529.49 ± 26.84	511.54 ± 18.75	551.28 ± 10.91	0.530
MG (mg/L)	126.66 ± 4.32	128.89 ± 2.89	129.79 ± 2.27	133.81 ± 10.60	0.861
HK (ng/L)	405.49 ± 18.84	426.08 ± 31.98	533.59 ± 61.46	490.46 ± 96.43	0.457
PK (ng/L)	492.36 ± 59.48	558.10 ± 35.08	628.01 ± 86.15	721.53 ± 22.74	0.093
CK (ng/mL)	81.58 ± 2.39 ^b^	94.50 ± 3.69 ^b^	90.08 ± 7.36 ^b^	116.05 ± 4.79 ^a^	0.003
MDH (pg/mL)	462.16 ± 29.52 ^b^	474.51 ± 22.23 ^b^	486.85 ± 47.30 ^b^	694.26 ± 51.86 ^a^	0.009
SDH (μmol/L)	368.95 ± 24.41 ^b^	424.81 ± 45.68 ^b^	372.04 ± 21.66 ^b^	557.22 ± 31.62 ^a^	0.010
LA (μg/L)	1406.13 ± 64.59 ^a^	1218.17 ± 23.92 ^a^	1318.83 ± 66.10 ^a^	963.17 ± 114.03 ^b^	0.015

Note: ^a,b^ Means with different superscripts in the same row are significantly different (*p* < 0.05). Glutathione peroxidase (GSH-PX), Superoxide dismutase (SOD), total antioxidant capacity (T-AOC), Catalase (CAT), Malondialdehyde (MDA), Immunoglobulin A (IgA), Immunoglobulin G (IgG), Immunoglobulin M (IgM), Tumor necrosis factor-α (TNF-α), Interleukin-1 beta (IL-1β), Interleukin 6 (IL-6), Lactate dehydrogenase (LDH), Glucose (GLU), Muscle glycogen (MG), Hexokinase (HK), Pyruvate Kinase (PK), Creatine Kinase (CK), Malate Dehydrogenase (MDH), Succinate dehydrogenase (SDH), and lactic acid (LA).

**Table 2:** Composition and nutrient level of the base ration (DM) %.

Items	MP
Corn	51.50
Soybean meal	2.00
Canola meal	12.80
Cottonseed meal	2.00
Palm meal	25.00
NaCl	1.00
Talcum powder	1.00
Baking soda	0.10
Premix (No. 6)	4.60
Total	100.00
DE/(MJ·kg^−1^)	12.84
Crude ash	12.13
Ether extract	3.44
Crude fiber	11.05
Neutral detergent fiber	26.04
Acid detergent fiber	19.11
Ca	0.84
*p*	0.40

Note: (1) Provided per kilogram of diets: Cu 15 mg, Fe 55 mg, Zn 25 mg, Mn 40 mg, Se 0.30 mg, I 0.5 mg, Co 0.20 mg, VA 20,000 IU, VD 4000 IU, and VE 40 IU.

**Table 3:** Primers used in qRT-PCR.

Name	Primer Sequence (5′-3′)	Tm (°C)	Product Length
Ncbi:101111913 *GLYCTK*	TGAGCGGCGGCACTGATG GAAGCGGCAGAAGAAGGTATGTG	60.0	149 bp
Ncbi:493968 *CYP17A1*	GCCCAGAGCAGGATTCAAAGC ACGAAGGATGGTGTAGCAGGTAG	60.0	135 bp
Ncbi:100192423 *ODC1*	GCCTGGACCGCATTGTTGAG AATCCATTGAAGGTAGAAGCAGCAG	60.0	115 bp
Ncbi:101118747 *GOT1*	GTGAGGAAGGTGGAGCAGAGG AGGCGGGAAGCACAGGTTC	60.0	101 bp
ncbi_101108953 *PDE4D*	TTCAGACAGTTGGAACAAGCAGAC TGGAGTCGGCAGAAGTGATAGC	60.0	112 bp
MSTRG.15268 *TAT*	GGAGTTCACGGAGCAGTTAGTTG GCCTCCAGCATCATCACTTCAG	60.0	120 bp
ncbi_101102876 *HNMT*	ATTCTAAGCATTGGCGGTGGTG GTTCAGCACTTGGTTCAACAACTTC	60.0	115 bp
ncbi_101108052 *HDC*	AGCAGACCTTCAGCGTAGACC AGGACCGAATCACGAACCAGAG	60.0	132 bp
MSTRG.17338 *HAL*	GACGACGCCCGCTTCCTC AACCACTTCCACGAACTCATTGTC	60.0	105 bp
ncbi_443489 *PFKM*	ATCCCTGCCACTGTCTCCAAC CTGCCGCTGACTGCTTGATG	60.0	112 bp

## Data Availability

The datasets presented in this study can be found in online repositories. The names of the repository/repositories and accession number(s) are NCBI SRA (accession: PRJNA1122402) and MetaboLights (accession: MTBLS10767).

## Supplementary Materials

The supporting information can be downloaded at: https://www.mdpi.com/article/10.3390/ijms25189865/s1.

## References

[1] Zhang X., Han L., Hou S., Raza S.H.A., Wang Z., Yang B., Sun S., Ding B., Gui L., Simal-Gandara J. (2022). Effects of different feeding regimes on muscle metabolism and its association with meat quality of Tibetan sheep. Food Chem..

[2] Zhou L., Raza S.H.A., Han L.J., Ma B.Y., Althobaiti F., Kesba H., Shukry M., Ghamry H.I., Gao Z.H., Hou S.Z. (2022). Effects of dietary concentrate: Forage ratio on development of gastrointestinal tract in black Tibetan sheep. J. Appl. Anim. Res..

[3] Gui L.S., Raza S.H.A., Allam F., Zhou L., Hou S.Z., Khan I., Kakar I.U., Abd El-Aziz A.H., Jia J.L., Sun Y.G. (2021). Altered milk yield and rumen microbial abundance in response to concentrate supplementation during the cold season in Tibetan sheep. Electron. J. Biotechnol..

[4] Jin S.J., Pang Q., Yang H., Diao X.P., Shan A.S., Feng X.J. (2021). Effects of dietary resveratrol supplementation on the chemical composition, oxidative stability and meat quality of ducks (*Anas platyrhynchos*). Food Chem..

[5] Zhang L., Dong M.N., Deng J., Zhang C.H., Liu M.W. (2022). Resveratrol exhibits neuroprotection against paraquat-induced PC12 cells via heme oxygenase 1 upregulation by decreasing MiR-136-5p expression. Bioengineered.

[6] Kim E.N., Lim J.H., Kim M.Y., Ban T.H., Jang I.A., Yoon H.E., Park C.W., Chang Y.S., Choi B.S. (2018). Resveratrol, an Nrf2 activator, ameliorates aging-related progressive renal injury. Aging.

[7] Vargas-Mendoza N., Morales-González Á., Madrigal-Santillán E.O., Madrigal-Bujaidar E., Álvarez-González I., García-Melo L.F., Anguiano-Robledo L., Fregoso-Aguilar T., Morales-Gonzalez J.A. (2019). Antioxidant and Adaptative Response Mediated by Nrf2 during Physical Exercise. Antioxidants.

[8] Meng Q., Li J., Wang C., Shan A. (2023). Biological function of resveratrol and its application in animal production: A review. J. Anim. Sci. Biotechnol..

[9] Zheng C.B., Song B., Duan Y.H., Zhong Y.Z., Yan Z.M., Zhang S.Y., Li F.N. (2020). Dietary β-hydroxy-β-methylbutyrate improves intestinal function in weaned piglets after lipopolysaccharide challenge. Nutrition.

[10] Liang Y., Zhou J., Ji K., Liu H., Degen A., Zhai M., Jiao D., Guo J., Zhao Z., Yang G. (2019). Protective Effect of Resveratrol Improves Systemic Inflammation Responses in LPS-Injected Lambs. Animal.

[11] Ryu C.H., Kim B.H., Lee S., Bang H.T., Baek Y.C. (2022). Effects of Supplemented Resveratrol on In Vitro Ruminal Fermentation and Growth Performance of Hanwoo Calves. Animal.

[12] Cebulska K., Sobiech P., Milewski S., Ząbek K. (2019). Efficacy of β-hydroxy-β-methylbutyric acid (HMB) for growing rate and its influence for health indicators in blood test of young early-weaning goats. Pol. J. Vet. Sci..

[13] Shu W., Yang M., Yang J., Lin S., Wei X., Xu X. (2022). Cellular crosstalk during liver regeneration: Unity in diversity. Cell Commun. Signal. CCS.

[14] Han H., Zhang T., Jin Z., Guo H., Wei X., Liu Y., Chen Q., He J. (2017). Blood glucose concentration and risk of liver cancer: Systematic review and meta-analysis of prospective studies. Oncotarget.

[15] Casey L.M., Hughes K.R., Saunders M.N., Miller S.D., Pearson R.M., Shea L.D. (2022). Mechanistic contributions of Kupffer cells and liver sinusoidal endothelial cells in nanoparticle-induced antigen-specific immune tolerance. Biomaterials.

[16] Hou Y.Q., Hu S.D., Li X.Y., He W.L., Wu G.Y., Wu G. (2020). Amino Acid Metabolism in the Liver: Nutritional and Physiological Significance. Amino Acids in Nutrition and Health: Amino Acids in Systems Function and Health.

[17] Li S., Tan H.Y., Wang N., Zhang Z.J., Lao L., Wong C.W., Feng Y. (2015). The Role of Oxidative Stress and Antioxidants in Liver Diseases. Int. J. Mol. Sci..

[18] Zheng M., Tian Z. (2019). Liver-Mediated Adaptive Immune Tolerance. Front. Immunol..

[19] Li C., Li X., Liu J., Fan X., You G., Zhao L., Zhou H., Li J., Lei H. (2018). Investigation of the differences between the Tibetan and Han populations in the hemoglobin-oxygen affinity of red blood cells and in the adaptation to high-altitude environments. Hematology.

[20] Konishi T., Yoshidome H., Shida T., Furukawa K., Takayashiki T., Kuboki S., Takano S., Miyazaki M., Ohtsuka M. (2021). Phosphorylated mTOR expression as a predictor of survival after liver resection for colorectal liver metastases. J. Surg. Oncol..

[21] Li S., Han B., Li J., Lv Z., Jiang H., Liu Y., Yang X., Lu J., Zhang Z. (2024). Resveratrol Alleviates Liver Fibrosis Induced by Long-Term Inorganic Mercury Exposure through Activating the Sirt1/PGC-1α Signaling Pathway. J. Agric. Food Chem..

[22] Gezer A., Ustundag H., Mendil A.S., Bedir G., Duysak L. (2024). Hepatoprotective effects of resveratrol on α-amanitin-induced liver toxicity in rats. Toxicon.

[23] Farías J.G., Herrera E.A., Carrasco-Pozo C., Sotomayor-Zárate R., Cruz G., Morales P., Castillo R.L. (2016). Pharmacological models and approaches for pathophysiological conditions associated with hypoxia and oxidative stress. Pharmacol. Ther..

[24] Sin T.K., Pei X.M., Teng B.T., Tam E.W., Yung B.Y., Siu P.M. (2013). Oxidative stress and DNA damage signalling in skeletal muscle in pressure-induced deep tissue injury. Pflügers Arch. Eur. J. Physiol..

[25] Kulczynski B., Sidor A., Gramza-Michalowska A. (2019). Characteristics of Selected Antioxidative and Bioactive Compounds in Meat and Animal Origin Products. Antioxidants.

[26] Zhang W.F., Gong M.Y., Zhang W.N., Mo J.T., Zhang S.M., Zhu Z., Wang X.N., Zhang B., Qian W.K., Wu Z. (2022). Thiostrepton induces ferroptosis in pancreatic cancer cells through STAT3/GPX4 signalling. Cell Death Dis..

[27] Jomova K., Alomar S.Y., Alwasel S.H., Nepovimova E., Kuca K., Valko M. (2024). Several lines of antioxidant defense against oxidative stress: Antioxidant enzymes, nanomaterials with multiple enzyme-mimicking activities, and low-molecular-weight antioxidants. Arch. Toxicol..

[28] Fu S., Lv R., Wang L., Hou H., Liu H., Shao S. (2018). Resveratrol, an antioxidant, protects spinal cord injury in rats by suppressing MAPK pathway. Saudi J. Biol. Sci..

[29] Sun Y.B., Geng S.X., Yuan T.Y., Liu Y., Zhang Y.X., Di Y.T., Li J.T., Zhang L.Y. (2021). Effects of Manganese Hydroxychloride on Growth Performance, Antioxidant Capacity, Tibia Parameters and Manganese Deposition of Broilers. Animals.

[30] Saber Y.H.A., Ibrahim S., Mahmoud K.G.M., Ahmed W.M., Ragab R.S.A., Seida A.A.M. (2024). Expression profile of viability and stress response genes as a result of resveratrol supplementation in vitrified and in vitro produced cattle embryos. Mol. Biol. Rep..

[31] Sen U. (2021). Maturation of bovine oocytes under low culture temperature decreased glutathione peroxidase activity of both oocytes and blastocysts. Pol. J. Vet. Sci..

[32] Kang Y.J., Song W., Lee S.J., Choi S.A., Chae S., Yoon B.R., Kim H.Y., Lee J.H., Kim C., Cho J.Y. (2024). Inhibition of BCAT1-mediated cytosolic leucine metabolism regulates Th17 responses via the mTORC1-HIF1α pathway. Exp. Mol. Med..

[33] Zheng J., Li B., Yan Y.T., Huang X.Y., Zhang E.P. (2023). β-Hydroxy-β-Methylbutyric Acid Promotes Repair of Sheep Myoblast Injury by Inhibiting IL-17/NF-κB Signaling. Int. J. Mol. Sci..

[34] Jiang D., Guo J., Liu Y., Li W., Lu D. (2023). Glycolysis: An emerging regulator of osteoarthritis. Front. Immunol..

[35] Rueda E.M., Johnson J.E., Giddabasappa A., Swaroop A., Brooks M.J., Sigel I., Chaney S.Y., Fox D.A. (2016). The cellular and compartmental profile of mouse retinal glycolysis, tricarboxylic acid cycle, oxidative phosphorylation, and ~P transferring kinases. Mol. Vis..

[36] Du F., Zhu X.H., Qiao H., Zhang X., Chen W. (2007). Efficient in vivo 31P magnetization transfer approach for noninvasively determining multiple kinetic parameters and metabolic fluxes of ATP metabolism in the human brain. Magn. Reson. Med..

[37] Frederiks W.M., Kümmerlin I.P., Bosch K.S., Vreeling-Sindelárová H., Jonker A., Van Noorden C.J. (2007). NADPH production by the pentose phosphate pathway in the zona fasciculata of rat adrenal gland. J. Histochem. Cytochem. Off. J. Histochem. Soc..

[38] Holloszy J.O., Oscai L.B. (1969). Effect of exercise on alpha-glycerophosphate dehydrogenase activity in skeletal muscle. Arch. Biochem. Biophys..

[39] Xu R., Yuan W., Wang Z. (2023). Advances in Glycolysis Metabolism of Atherosclerosis. J. Cardiovasc. Transl. Res..

[40] Gomez L.S., Zancan P., Marcondes M.C., Ramos-Santos L., Meyer-Fernandes J.R., Sola-Penna M., Da Silva D. (2013). Resveratrol decreases breast cancer cell viability and glucose metabolism by inhibiting 6-phosphofructo-1-kinase. Biochimie.

[41] Wan H., Zhu J., Wu C., Zhou P., Shen Y., Lin Y., Xu S., Che L., Feng B., Li J. (2017). Transfer of β-hydroxy-β-methylbutyrate from sows to their offspring and its impact on muscle fiber type transformation and performance in pigs. J. Anim. Sci. Biotechnol..

[42] Díaz-Piña D.A., Rivera-Ramírez N., García-López G., Díaz N.F., Molina-Hernández A. (2024). Calcium and Neural Stem Cell Proliferation. Int. J. Mol. Sci..

[43] He D., Guo Z., Pu J.L., Zheng D.F., Wei X.F., Liu R., Tang C.Y., Wu Z.J. (2016). Resveratrol preconditioning protects hepatocytes against hepatic ischemia reperfusion injury via Toll-like receptor 4/nuclear factor-κB signaling pathway in vitro and in vivo. Int. Immunopharmacol..

[44] Fang M., Feng C., Zhao Y.X., Liu X.Y. (2014). Camk2b protects neurons from homocysteine-induced apoptosis with the involvement of HIF-1α signal pathway. Int. J. Clin. Exp. Med..

[45] Messai Y., Noman M.Z., Hasmim M., Janji B., Tittarelli A., Boutet M., Baud V., Viry E., Billot K., Nanbakhsh A. (2014). ITPR1 protects renal cancer cells against natural killer cells by inducing autophagy. Cancer Res..

[46] Hochman A., Sternin H., Gorodin S., Korsmeyer S., Ziv I., Melamed E., Offen D. (1998). Enhanced oxidative stress and altered antioxidants in brains of Bcl-2-deficient mice. J. Neurochem..

[47] Yang B., Li H., Qiao Y., Zhou Q., Chen S., Yin D., He H., He M. (2019). Tetramethylpyrazine Attenuates the Endotheliotoxicity and the Mitochondrial Dysfunction by Doxorubicin via 14-3-3γ/Bcl-2. Oxidative Med. Cell. Longev..

[48] Palmes D., Spiegel H.U. (2004). Animal models of liver regeneration. Biomaterials.

[49] Shen X., Li X., Jia C., Li J., Chen S., Gao B., Liang W., Zhang L. (2023). HPLC-MS-based untargeted metabolomic analysis of differential plasma metabolites and their associated metabolic pathways in reproductively anosmic black porgy, Acanthopagrus schlegelii. Comp. Biochem. Physiol. Part D Genom. Proteom..

[50] Wang Q., Xu Z., Ai Q. (2021). Arginine metabolism and its functions in growth, nutrient utilization, and immunonutrition of fish. Anim. Nutr. (Zhongguo Xu Mu Shou Yi Xue Hui).

[51] Liu J., Liu Q., Han J., Feng J., Guo T., Li Z., Min F., Jin R., Peng X. (2021). N-Acetylcysteine Inhibits Patulin-Induced Apoptosis by Affecting ROS-Mediated Oxidative Damage Pathway. Toxins.

[52] Gill A.J., Kolson D.L. (2013). Dimethyl fumarate modulation of immune and antioxidant responses: Application to HIV therapy. Crit. Rev. Immunol..

[53] Beardsley R.E. (1962). Amino acid cross resistance in Agrobacterium tumefaciens. J. Bacteriol..

[54] Deshpande P.D., Harper A.E., Elvehjem C.A. (1957). Nutritional improvement of white flour with protein and amino acid supplements. J. Nutr..

[55] Ma Y., Cai G., Chen J., Yang X., Hua G., Han D., Li X., Feng D., Deng X. (2024). Combined transcriptome and metabolome analysis reveals breed-specific regulatory mechanisms in Dorper and Tan sheep. BMC Genom..

[56] Coggins A.J., Powner M.W. (2017). Prebiotic synthesis of phosphoenol pyruvate by α-phosphorylation-controlled triose glycolysis. Nat. Chem..

[57] Anderson N.M., Mucka P., Kern J.G., Feng H. (2018). The emerging role and targetability of the TCA cycle in cancer metabolism. Protein Cell.

[58] Wang C., Hao J., Liu X., Li C., Yuan X., Lee R.J., Bai T., Wang D. (2020). Isoforsythiaside Attenuates Alzheimer’s Disease via Regulating Mitochondrial Function Through the PI3K/AKT Pathway. Int. J. Mol. Sci..

[59] Shi Q., Zeng Y., Xue C., Chu Q., Yuan X., Li L. (2024). Development of a promising PPAR signaling pathway-related prognostic prediction model for hepatocellular carcinoma. Sci. Rep..

[60] Ramanarayanan P., Heine G., Worm M. (2023). Vitamin A and vitamin D induced nuclear hormone receptor activation and its impact on B cell differentiation and immunoglobulin production. Immunol. Lett..

[61] Heine G., Hollstein T., Treptow S., Radbruch A., Worm M. (2018). 9-cis retinoic acid modulates the type I allergic immune response. J. Allergy Clin. Immunol..

[62] Zhang X., Chen L., Hardwick J.P. (2000). Promoter activity and regulation of the CYP4F2 leukotriene B(4) omega-hydroxylase gene by peroxisomal proliferators and retinoic acid in HepG2 cells. Arch. Biochem. Biophys..

[63] Zhang L., Zhang H., Gu J., Xu W., Yuan N., Sun J., Li H. (2022). Glabridin inhibits liver fibrosis and hepatic stellate cells activation through suppression of inflammation and oxidative stress by activating PPARγ in carbon tetrachloride-treated mice. Int. Immunopharmacol..

[64] Landini L., Dadson P., Gallo F., Honka M.J., Cena H. (2023). Microbiota in anorexia nervosa: Potential for treatment. Nutr. Res. Rev..

[65] Kim D., Langmead B., Salzberg S.L. (2015). HISAT: A fast spliced aligner with low memory requirements. Nat. Methods.

[66] Love M.I., Huber W., Anders S. (2014). Moderated estimation of fold change and dispersion for RNA-seq data with DESeq2. Genome Biol..

