# Supplementary material for: Immune, Oxidative, and Morphological Changes in the Livers of Tibetan Sheep after Feeding Resveratrol and β-Hydroxy-β-methyl Butyric Acid: A Transcriptome–Metabolome Integrative Analysis
Source: Int J Mol Sci. 2024 Sep 12;25(18):9865. doi: 10.3390/ijms25189865 (PMC11432669; doi:10.3390/ijms25189865)
Supplement: Supplementary file 1 [file ijms-25-09865-s001.zip › Table S1.pdf]

Sample	Clean reads	Total reads	Unmapped （%）	Unique Mapped （%）	Multiple Mapped （%）	Total Mapped （%）
H1	373,808,20	372,863,80	1836261 (4.92%)	33067266 (88.68%)	2382853 (6.39%)	35450119 (95.08%)
H2	411,036,94	409,952,08	1912785 (4.67%)	36464306 (88.95%)	2618117 (6.39%)	39082423 (95.33%)
H3	415,758,60	414,576,54	2021018 (4.87%)	36687450 (88.49%)	2749186 (6.63%)	39436636 (95.13%)
H4	372,443,94	371,444,26	1694746 (4.56%)	33065977 (89.02%)	2383703 (6.42%)	35449680 (95.44%)
H-RES-1	378,295,20	377,117,78	1922310 (5.10%)	32508424 (86.20%)	3281044 (8.70%)	35789468 (94.90%)
H-RES-2	366,836,72	365,571,36	2002217 (5.48%)	31340736 (85.73%)	3214183 (8.79%)	34554919 (94.52%)
H-RES-3	360,179,88	358,728,66	1968576 (5.49%)	30722499 (85.64%)	3181791 (8.87%)	33904290 (94.51%)
H-RES-4	388,190,10	386,721,22	2290166 (5.92%)	32977863 (85.28%)	3404093 (8.80%)	36381956 (94.08%)
H-HMB-1	412,452,22	408,612,26	2720671 (6.66%)	34337811 (84.04%)	3802744 (9.31%)	38140555 (93.34%)
H-HMB-2	395,215,84	393,572,06	1997697 (5.08%)	33924518 (86.20%)	3434991 (8.73%)	37359509 (94.92%)
H-HMB-3	361,653,82	360,073,56	1878825 (5.22%)	30988376 (86.06%)	3140155 (8.72%)	34128531 (94.78%)
H-HMB-4	414,619,22	412,694,04	1905020 (4.62%)	35644236 (86.37%)	3720148 (9.01%)	39364384 (95.38%)
H-RES-HMB-1	400,283,44	399,250,44	1901247 (4.76%)	35293588 (88.40%)	2730209 (6.84%)	38023797 (95.24%)
H-RES-HMB-2	404,301,22	401,876,88	1805531 (4.49%)	35522342 (88.39%)	2859815 (7.12%)	38382157 (95.51%)
H-RES-HMB-3	375,231,42	374,331,22	1970905 (5.27%)	32897856 (87.88%)	2564361 (6.85%)	35462217 (94.73%)
H-RES-HMB-4	394,169,64	393,233,92	1800160 (4.58%)	34761001 (88.40%)	2762231 (7.02%)	37523232 (95.42%)
